# Translation Initiation Control of RNase E-Mediated Decay of Polycistronic *gal* mRNA

**DOI:** 10.3389/fmolb.2020.586413

**Published:** 2020-11-06

**Authors:** Heung Jin Jeon, Changjo Kang, Monford Paul Abishek N, Yonho Lee, Xun Wang, Dhruba K. Chattoraj, Heon M. Lim

**Affiliations:** ^1^Department of Biological Sciences, College of Biological Sciences and Biotechnology, Chungnam National University, Daejeon, South Korea; ^2^State Key Laboratory of Agricultural Microbiology, College of Life Science and Technology, Huazhong Agricultural University, Wuhan, China; ^3^Basic Research Laboratory, Center for Cancer Research, National Cancer Institute, Bethesda, MD, United States

**Keywords:** mRNA decay, RNase E, mRNA stability, translation initiation, polycistronic gal mRNA

## Abstract

In bacteria, mRNA decay is a major mechanism for regulating gene expression. In *Escherichia coli*, mRNA decay initiates with endonucleolytic cleavage by RNase E. Translating ribosomes impede RNase E cleavage, thus providing stability to mRNA. In transcripts containing multiple cistrons, the translation of each cistron initiates separately. The effect of internal translation initiations on the decay of polycistronic transcripts remains unknown, which we have investigated here using the four-cistron *galETKM* transcript. We find that RNase E cleaves a few nucleotides (14–36) upstream of the translation initiation site of each cistron, generating decay intermediates *galTKM*, *galKM*, and *galM* mRNA with fewer but full cistrons. Blocking translation initiation reduced stability, particularly of the mutated cistrons and when they were the 5′-most cistrons. This indicates that, together with translation failure, the location of the cistron is important for its elimination. The instability of the 5′-most cistron did not propagate to the downstream cistrons, possibly due to translation initiation there. Cistron elimination from the 5′ end was not always sequential, indicating that RNase E can also directly access a ribosome-free internal cistron. The finding in *gal* operon of mRNA decay by cistron elimination appears common in *E. coli* and *Salmonella*.

## Introduction

In bacteria, mRNA concentration is modulated to cope with a rapidly changing environment. The modulation is mediated by varying the synthesis as well as the decay rates of mRNA. In *Escherichia coli*, mRNA decay is primarily initiated by the endoribonuclease RNase E (Ono and Kuwano, 1979; Mudd et al., 1990; Babitzke and Kushner, 1991; Melefors and von Gabain, 1991; Taraseviciene et al., 1991; Carpousis et al., 2009). RNase E can target mRNAs for the initial cleavage either by first sensing a 5′ monophosphate group (5′ end-dependent pathway) or by bypassing this requirement (direct access pathway) (Mackie, 2013; Hui et al., 2014).

The 5′ end-dependent pathway begins with RNase E binding to the mono-phosphorylated 5′ end of transcripts (Celesnik et al., 2007) and cleavage at an internal site rich in AU sequences (McDowall et al., 1994). The N-terminal sensor domain of RNase E allows it to bind preferentially to the mono-phosphorylated rather than to the tri- or di-phosphorylated 5′ end of mRNA (Mackie, 1998; Jiang and Belasco, 2004; Callaghan et al., 2005). Thus, the 5′ end-dependent access requires prior conversion of transcripts with 5′ tri- or di-phosphorylated end to mono-phosphorylated end by RNA pyrophosphohydrolase (RppH) (Celesnik et al., 2007; Deana et al., 2008). RNase E can also bypass the 5′ end binding and access a cleavage site internal to the message directly (Joyce and Dreyfus, 1998; Baker and Mackie, 2003). In either case, RNase E cleavage results in the generation of the 5′ portion of mRNA with a new 3′-OH end and the 3′ portion of mRNA with a new 5′ end. Generally, the 5′ portion mRNA is degraded immediately by 3′→ 5′ exonuclease digestion (Mott et al., 1985; Belasco et al., 1986; Hui et al., 2014; Wang et al., 2019). The 3′ portion mRNA could be subjected to decay by further rounds of cleavage by RNase E by either of the pathways (Joyce and Dreyfus, 1998; Spickler et al., 2001; Hui et al., 2014).

There are several mechanisms that modulate RNase E activity. Secondary structures at the 5′ end can extend the stability of mRNA because these structures interfere with RNase E initial cleavage (Bouvet and Belasco, 1992; Emory et al., 1992). Translation has a substantial effect on mRNA decay since translating ribosomes tend to inhibit RNase E cleavage (Braun et al., 1998; Deana and Belasco, 2005; Dreyfus, 2009). By the same token, translation initiation also promotes the stability of mRNA (Arnold et al., 1998; Joyce and Dreyfus, 1998; Baker and Mackie, 2003). In view of these modulating factors, it is unsurprising that polycistronic mRNAs show a differential decay of component cistrons (Belasco et al., 1985; Newbury et al., 1987; Adhya, 2003; Dar and Sorek, 2018).

**FIGURE 1:**
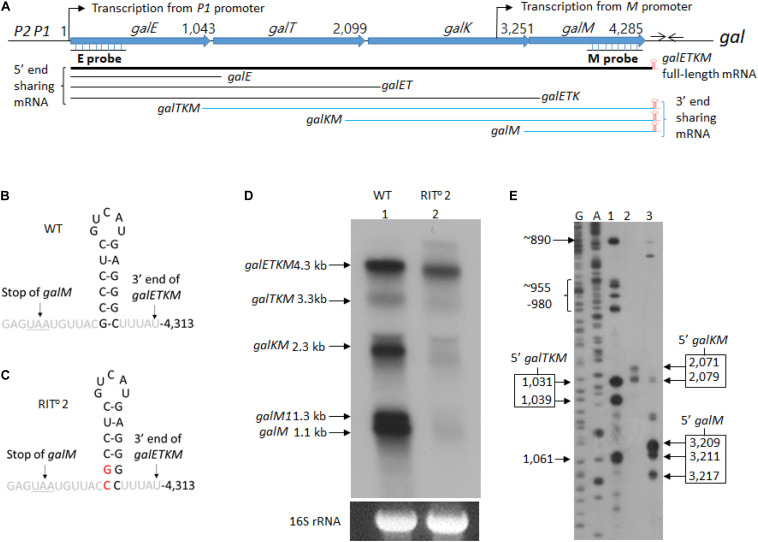
mRNA intermediates of the *gal* operon that share the 3′ end with the full-length *gal* mRNA. **(A)** Map of *galETKM* operon comprising the genes *galE*, *galT*, *galK*, and *galM* (Lewis and Adhya, 2015). Numbers indicate *gal* nucleotide residue coordinate, where 1 is the start of transcription from the *galP1* promoter. The number at the end of each gene belongs to the last nucleotide of the stop codon of that gene. The *gal* operon transcription from the *M* promoter initiates at 3,073. At six nucleotides downstream of the stop codon of *galM* gene, there is a 17-nucleotide inverted repeat sequence (head-to-head arrows). It forms the terminator hairpin (red) that terminates transcription and protects *galETKM* mRNA from 3′→5′ exonuclease digestion (McLaren et al., 1991; Wang et al., 2019). Two probes, E and M (underlined), were used to identify intermediates that share the 5′ and the 3′ ends with the full-length *galETKM* mRNA. The 3′-end-sharing mRNA shares the same 3′ end at 4,313 and have 5′ ends at the beginning of each comprising gene. **(B)** Nucleotide sequence of the terminator hairpin. **(C)** Nucleotide sequence of the terminator hairpin in the RIT^0^2 *gal* mutant. The nucleotide changes are indicated in red. **(D)** Northern blot of wild type (WT; lane 1) and RIT^0^2 *gal* mutant (lane 2). *gal* mRNA prepared from cells grown up to OD_600_ of 0.6 in Luria broth with 0.5% galactose. The blot was probed with the M probe complementary to the 3′ half of the *galM* gene of the *gal* operon **(A)**. **(E)** 5′RACE of WT *gal* mRNA prepared under the same condition as in **(D)**. The lanes show the 5′ ends found near the beginning of either *galT* (lane 1) or *galK* (lane 2) or *galM* (lane 3) genes. DNA sequencing ladders used as molecular weight markers are in lanes marked G and A. Note that the 5′RACE procedure depends on a ligation step that requires a mono-phosphorylated 5′ end. Thus, 5′RACE detects specifically 5′ ends of mRNA that are mono-phosphorylated.

Several models to account for the decay process of polycistronic mRNA in relation to translating ribosomes have been proposed (Alifano et al., 1994a). In polycistronic mRNA, the decay process is expected to be complex because, other than the secondary structures on RNA that interfere with RNase E and/or exoribonuclease activities, translation initiation at the internal start sites of each of the comprising cistrons could also be a compounding factor. Here we have addressed the decay process of a polycistronic mRNA in relation to translation initiation using the well-studied *gal* operon of *E. coli*. The operon harbors four genes–*galE*, *galT*, *galK*, and *galM* (about 1 kb each) – and produces the full-length mRNA *galETKM* of 4.3-kb size with the structure: 5′-g*alE–galT–galK–galM-*terminator hairpin*-*3′ (Figure 1A; Adhya, 2003). The terminator hairpin causes Rho-independent transcription termination and blocks 3′→ 5′ exoribonuclease digestion initiated from the free 3′-OH end of the transcript, providing stability to the *galETKM* mRNA (Figure 1B; Wang et al., 2019). Interestingly, the operon also produces two other mRNA species, *galETK* and *galTKM*, both about 3.3 kb. The *galETK* mRNA, 5′-g*alE–galT–galK-*3′, is generated by Rho-dependent transcription termination (Lee et al., 2008; Wang et al., 2014, 2015). How the *galTKM* mRNA, 5′-*galT–galK–galM-*3′, is generated is not clear.

In *E. coli*, mRNA decay is generally believed to proceed in 5′ to 3′ direction without any known 5′ to 3′ exoribonuclease (Apirion, 1973). There are also a number of well-documented examples of mRNAs whose degradation begins with cleavage far from the 5′ end (von Gabain et al., 1983; Belasco et al., 1985; Newbury et al., 1987; Braun et al., 1998). In addition to *galTKM* mRNA, the *gal* operon also produces *galKM* (formerly known as mK2; Lee et al., 2008). Most intermediate *gal* mRNA species are stable enough to be detected by northern blot and harbor full open reading frames (ORF) of the remaining cistrons. In other words, *gal* operon produces mRNA species that are cistron(s)-shorter from the 5′ end of the full-length mRNA. Here we have addressed whether the intermediates *galTKM* and *galKM* mRNA are generated by cistron elimination of *galETKM* from the 5′ end.

In this study, using northern blot and 5′RACE assays, we found that the decay of polycistronic *galETKM* mRNA by and large proceeds by eliminating nearly full-length cistron(s) from the 5′ end, generating *galTKM*, *galKM*, and *galM* mRNA species as decay intermediates, but the elimination is not always sequential. Translation initiation at the newly generated 5′ end of the intermediate mRNA contributes to the stability of the decay intermediates. A sequence analysis of the 5′ end of mRNA generated as a result of RNase E cleavage (Chao et al., 2017) shows that eliminating cistrons from the 5′ end is the common theme in polycistronic mRNA decay in *E. coli* as well as in *Salmonella*.

## Materials and Methods

### Extraction of RNA From *E. coli* Cells

Total RNA was prepared from 2 × 10^8^ *E. coli* cells grown to OD_600_ of 0.6 in Luria broth (LB) as described previously (Wang et al., 2014). The primers used in this study are listed in Supplementary Table S1.

### 5′RACE

To determine the 5′ end of the *gal* mRNA, we ligated the 3′ end of the 5S rRNA in the RNA preparation from *E. coli* cells to 5′ ends of RNA in the same RNA preparation (Lee et al., 2008). Later, the *gal* mRNA of interest was amplified using RT-PCR. First, RNA ligation reaction was performed in a 15-μl volume containing 2.5 μg of total RNA, 5 U T4 RNA ligase (Ambion, United States), and 10 U rRNasin (Promega, United States) at 37°C for 3 h. One microgram of the ligated RNA was reverse-transcribed at 37°C for 2 h in a 20-μl reaction volume containing 4 U reverse transcriptase (Qiagen, Germany), 0.5 mM each of dNTP, 10 μM random hexamer primer (Takara, Japan), and 10 U rRNasin. Two microliters of the reverse transcription reaction was used as template for PCR amplification of the *gal* cDNA of interest in the next step in a total volume of 20 μl using 1 U of HotStar *Taq* DNA polymerase (Qiagen, Germany), with a forward primer complementary to the 3′ end of the *E. coli* 5S rRNA and a reverse primer specific to a *gal* cDNA of interest (Lee et al., 2008) (listed in Supplementary Table S1). The 5′ end of the *gal* cDNA (thus, *gal* mRNA) of interest was assayed by extending a ^32^P-labeled DNA primer bound to a specific region of the amplified *gal* cDNA of interest. This “primer extension” reaction was performed in a 20-μl volume containing 10 μl of amplified *gal* cDNA reaction, 0.75 μl ^32^P-labeled primer, 0.15 mM each of dNTP, and 1 U *Taq* polymerase with 25 cycles of amplification. The exact location of the 5′ ends of the *gal* mRNA was identified as follows: The extended-primer DNAs were resolved on 8% polyacrylamide–urea sequencing gel, and radioactive bands were visualized on an X-ray film. We measured the number of nucleotides of the extended-primer DNAs using the DNA sequencing ladder as a ruler. By subtracting the number of nucleotides in the primer DNA from the number of nucleotides of the extended-primer DNA, we located the exact position of the 5′ end of the *gal* mRNA in the nucleotide residue coordinates of the *gal* operon.

### Northern Blot

Typically, 10 μg of total RNA (after staining with 1 μg/ml ethidium bromide) was resolved by electrophoresis using 1.2% (wt/vol) formaldehyde–agarose gel at 5 V/cm for 4 h. RNA on the gel was then transferred overnight to a positively charged nylon membrane (Ambion, United States) using a downward transfer system (TurboBlotter, Whatman, United Kingdom). The nylon membrane was baked at 80°C for 1 h. M3 probe was prepared by PCR using a pair of primers complementary to 3,751 and 4,285 in *gal* coordinates. Probe DNA was labeled with ^32^P. Hybridization procedures followed the manufacturer’s instructions (Ambion, United States). RNA bands were quantified using the software JMOL (NIH).

## Results

### mRNA Intermediates of the *gal* Operon That Share the 3′ End With the Full-Length *gal* mRNA

We performed northern blot of total RNA prepared from wild-type *E. coli* cells (MG1655) grown in LB supplemented with 0.5% galactose to OD_600_ of 0.6. The blots were analyzed using the M-probe, which hybridizes to the distal half of the last gene of the *gal* operon, *galM* (Figure 1A). We detected five mRNA species with sizes of 4.3, 3.3, 2.3, 1.3, and 1.1 kb (lane 1 in Figure 1D). We named these mRNA species *galETKM*, *galTKM*, *galKM, galM1*, and *galM*, respectively, as we show later that these mRNA species have their 5′ ends at the beginning of each cistron but have the same 3′ end, which is at the end of the operon (Figure 1A). We called these mRNA species “3′-end-sharing” mRNA.

The 1.3-kb mRNA named *galM1* turned out to be the result of transcription initiation at an internal promoter in front of the *galM* gene, which terminated at the end of *galM* (Supplementary Figure S1). We designated the internal promoter as *M* promoter. We excluded the *galM1* and *galETKM* mRNA species from the 3′-end-sharing group of decay intermediates because these are primary transcripts (see below).

To test if the 3′-end-sharing mRNA species harbor the terminator hairpin structure of the full-length *galETKM* 3′ end (Figures 1A,B; Wang et al., 2019), we performed northern blot on RNA prepared from the culture of MG1655Δ*gal* strain, where the chromosomal *gal* operon is entirely deleted. The strain harbored a pBAC-derived low-copy-number plasmid that carries a mutant version of the *gal* operon, RIT^o^2 (Wang et al., 2014). In the RIT^o^2 mutant, two bases from the bottom of the stem of the terminator hairpin are substituted to their complementary bases (Figure 1C). In the RIT^o^2 mutant, the function of the terminator hairpin in the blockage of 3′→ 5′ exoribonuclease was impaired (Wang et al., 2019). When RNA from the RIT^o^2 mutant was analyzed using the M-probe, we confirmed that the mutations caused the shortening of the *galETKM* mRNA by about 100 nucleotides and also decreased the *galETKM* amount to about 40% of the wild type (WT; lane 2 in Figure 1D; Wang et al., 2019). The northern blot also showed that *galTKM*, galKM, and *galM1* also got similarly shorter than their corresponding WT counterparts (lane 2, Figure 1D), and the amount of these mRNA species decreased to less than 10% of their amounts in the WT. The *galM* mRNA was hardly observed in the RIT^o^2 mutant (lane 2, Figure 1D). These results demonstrate that the 3′-end-sharing mRNA species and the primary transcript *galM1* harbor the terminator hairpin structure of the full-length *galETKM*.

To determine the 5′ end sequences of the 3′-end-sharing mRNA, we used 5′RACE. The assay showed that the 5′ ends of the *galTKM* mRNA were clustered around *gal* coordinates 1,031 and 1,039 (lane 1, Figure 1E), those of the *galKM* mRNA at 2,071 and 2,079 (lane 2, Figure 1E), and those of the *galM* mRNA at 3,209, 3,211, and 3,217 (lane 3, Figure 1E). The *gal* nucleotide coordinates, starting from the *P1*-transcription initiation site, are as in Figure 1A. These results indicate that the 5′ ends of the 3′-end-sharing mRNA are generated by endoribonucleolytic cleavages occurring near cistron junctions, mostly within the last few codons of the respective upstream ORF.

### The 3′-End-Sharing mRNA Species Are the Intermediate Products of the RNase E-Mediated Decay of *galETKM*

**FIGURE 2:**
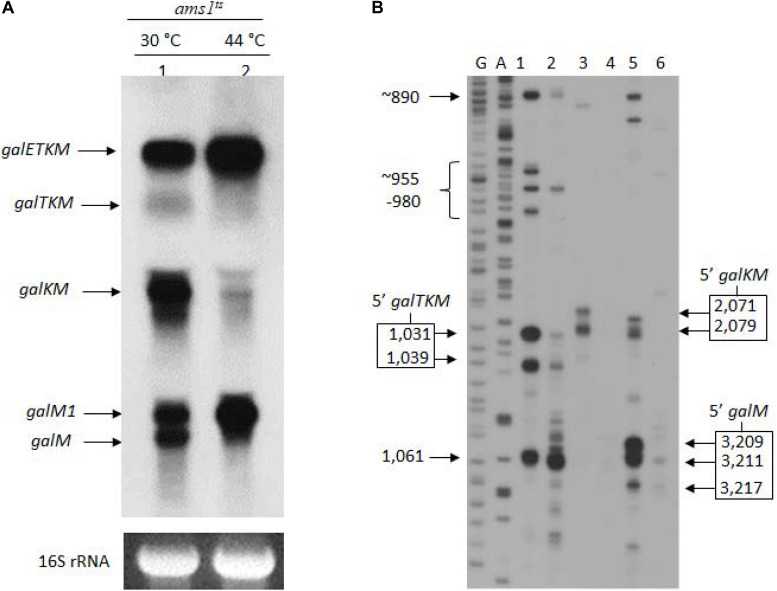
The 3′-end-sharing mRNA species are the intermediate products of the RNase E-mediated decay of *galETKM*. **(A)** Northern blot of the *gal* mRNA in GW20 (*ams1*^*ts*^) cells grown at either the permissive temperature of 30°C (lane 1) or the non-permissive temperature of 44°C (lane 2). The blot was probed with the M probe complementary to the 3′ half of the *galM* gene of the *gal* operon. *galETKM* and *galM1* are the primary transcripts, and *galTKM*, *galKM*, and *galM* are the decay intermediates. **(B)** 5′RACE of *gal* mRNA prepared from GW20 (*ams1*^*ts*^) cells as in **(A)**. The lanes show the 5′ ends of RNA at the permissive and the non-permissive temperatures found near the beginning of either the *galT* gene (lanes 1 and 2) or the *galK* gene (lanes 3 and 4) or the *galM* gene (lanes 5 and 6). DNA sequencing ladders used as molecular weight markers are in lanes marked G and A. An exception was the 5′ end at 1,061, which was unchanged at the two temperatures, indicating that this 5′ end was not generated by RNase E.

RNase E is the major endoribonuclease in *E. coli*. To test whether this nuclease is responsible for cleavages near cistron junctions, generating the 3′-end-sharing mRNA species, we assayed for their presence in GW20 (*ams1*^*ts*^), a strain temperature-sensitive for RNase E activity (Wachi et al., 1997). We subjected the total RNA prepared from GW20 to northern blot analysis. Cells were grown to OD_600_ of 0.2 at 30°C in LB supplemented with 0.5% galactose, and the culture was divided into two halves. One half was shifted to 44°C, a non-permissive temperature, whereas the other half continued to grow at 30°C until they reached OD_600_ of 0.6. The northern blot results showed that, at non-permissive temperature, when GW20 cells were deprived of functional RNase E, *galTKM*, *galKM*, and *galM* mRNA species decreased drastically (Figure 2A). These results support the view that the 3′-end-sharing mRNA species, *galTKM*, *galKM*, and *galM*, are the products of RNase E cleavage.

To confirm that the 5′ ends of the 3′-end-sharing mRNA (shown in Figure 1E) are generated by RNase E cleavage, we assayed the 5′ ends by 5′RACE on total RNA from GW20 cells grown at permissive and non-permissive temperatures. The results showed that, at the non-permissive temperature of 44°C, most 5′ ends of *galTKM* (lane 2, Figure 2B), *galKM* (lane 4, Figure 2B), and *galM* (lane 6, Figure 2B) were less than 10% of their amounts at the permissive temperature of 30°C (lanes 1, 3, and 5, Figure 2B). These results demonstrate that the 5′ ends of the 3′-end-sharing mRNA species are generated primarily by RNase E cleavage.

It is noteworthy that, in contrast to the decreasing amounts of 3′-end-sharing mRNA at the non-permissive temperature, the level of the primary transcripts, *galETKM* and *galM1*, increased by about twofold (lane 2, Figure 2A). The increased level of primary transcripts is to be expected if the 3′-end-sharing mRNA species are the RNase E-mediated decay products of *galETKM*.

### The 5′ Portion mRNA Generated by RNase E Cleavage Is Degraded Rapidly

So far, we have analyzed only the 3′ portions of the *galETKM* mRNA after the RNase E cleavages at 1,031 and 1,039, 2,071 and 2,079, and 3,209, 3,211, and 3,217 that generated the 3′-end-sharing mRNA species *galTKM*, *galKM*, and *galM*, respectively. The 5′ portion of the *galETKM* mRNA after the RNase E cleavages should be in sizes of 1,031–1,039, 2,071–2,079, and 3,209–3,217 nucleotides long, respectively.

**FIGURE 3:**
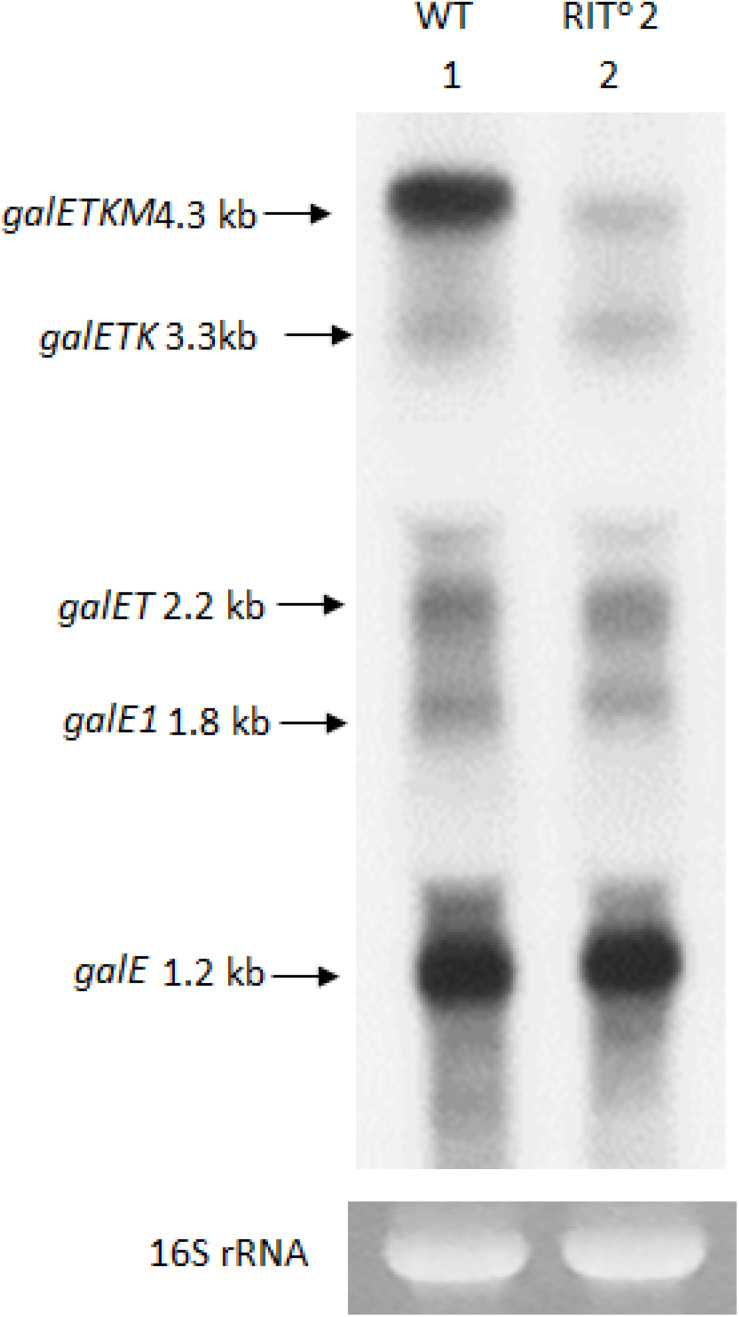
The 5′ portion mRNA generated by RNase E cleavage is degraded rapidly. Northern blot of *gal* mRNA probed with the E probe. *gal* mRNA was prepared from wild-type cells (lane 1), and the RIT^0^2 *gal* mutant cells (lane 2) were grown up to OD_600_ of 0.6 in Luria broth with 0.5% galactose. The blot was probed with the E probe complementary to the 3′ half of the *galE* gene of the *gal* operon (Figure 1A).

To detect these species, we performed northern blot of *gal* mRNA from wild-type and RIT^o^2 mutant cells, this time using the E-probe (Figure 1A) that hybridizes to the first half of the *galE* gene (Figure 1A). The northern blot of the wild-type cells showed *galETKM* (4.3 kb), *galETK* (3.3 kb), *galET* (2.2 kb), *galE1* (1.8 kb), and *galE* (1.2 kb) (Figure 3). These have been presented as “5′-end-sharing” mRNA in our previous reports (Figure 1A; Lee et al., 2008; Wang et al., 2014, 2015). In the RIT^o^2 mutant, where the terminator hairpin is no longer functioning (Wang et al., 2019), *galETKM* is the only mRNA species affected; *galETKM* reduced in size by ∼100 nucleotides and decreased in amount to about 25% of the WT, but all the other mRNA species were present as much as they were in wild-type cells (Figure 3). These results demonstrate that the *galETKM* mRNA is *not* a precursor of all other *gal* mRNA species shown in Figure 3. These mRNA species are known to be generated as a result of premature transcription termination by Rho (Adhya, 2003; Wang et al., 2015). Indeed bicyclomycin (BCM), the Rho-inhibitor, inhibited the production of all the 5′-sharing mRNA species (Supplementary Figure S2).

These results thus suggest that the 5′ portion of the *galETKM* mRNA after the RNase E cleavages is removed immediately, possibly by 3′→5′ exoribonucleases, as has been demonstrated in many previous studies (Mott et al., 1985; Belasco et al., 1986; Hui et al., 2014; Wang et al., 2019).

### Temporal Order of Cistron Elimination From the *galETKM* mRNA

To test whether the cistrons are eliminated one at a time from the 5′ end of the *galETKM* mRNA, we performed a “temperature shift-down” (TSD) experiment and followed the order of appearance of the decay intermediates with time. We used the GW20 (*ams1*^*ts*^) strain, expecting that RNase E would regain its function and initiate the decay process as the temperature of the GW20 culture is brought down from 44 to 30°C. If the cistrons are eliminated one at a time from the 5′ end of *galETKM*, we expect that the order of appearance of the intermediates would be *galTKM*, *galKM*, and finally *galM*.

**FIGURE 4:**
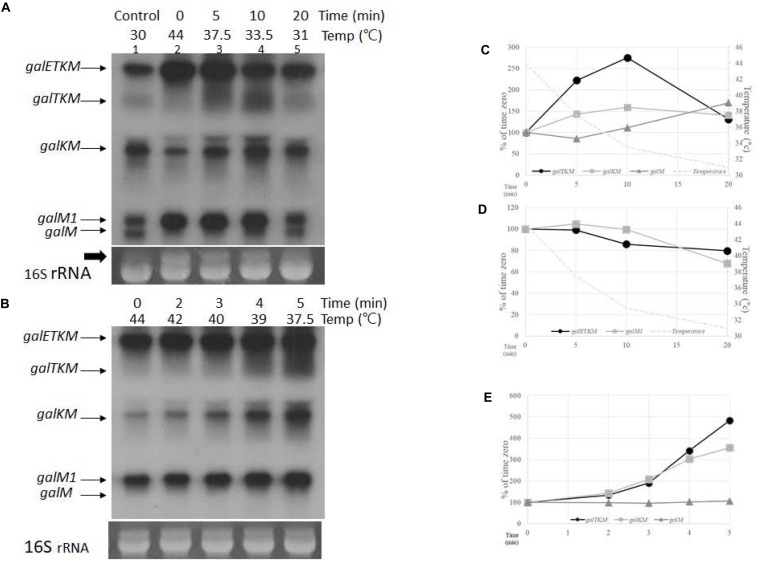
Temporal order of cistron elimination from the *galETKM* mRNA. **(A)** Northern blot (M-probed) of *gal* mRNA prepared from GW20 (*ams1*^*ts*^) cells taken at various times after temperature shift-down. The culture of GW20 cells grown up to *A*_600_ of 0.6 in Luria broth (LB) at 44°C was transferred to 30°C, and growth was allowed to continue. Aliquots of cells were taken at 0, 5, 10, and 20 min after transfer (lanes 2–5). The temperature of the culture at each time point is indicated. Lane 1 is *gal* mRNA from GW20 cells grown continuously to *A*_600_ of 0.6 in LB at 30°C. The arrow indicates the putative 17S rRNA, a precursor of 16S rRNA (Li et al., 1999), which were used here as loading controls. **(B)** Northern blot (M-probed) of *gal* mRNA from GW20 cells taken at 0, 2, 3, 4, and 5 min after temperature shift-down from 44 to 30°C. Temperature of the culture at each time point is indicated. **(C)** Changes in the amount of decay intermediate mRNA species after 5, 10, and 20 min of temperature shift-down. Amounts relative to that of time-zero were graphed; *galTKM* (dark circle), *galKM* (gray square), and *galM* (gray triangle). Temperature changes are indicated by a dotted line. **(D)** Changes in the amount of primary transcripts after 5, 10, and 20 min of temperature shift-down. Amounts relative to that of time-zero were graphed; *galETKM* (dark circle) and *galM1* (gray square). Temperature changes are indicated by a dotted line. **(E)** Changes in the amount of decay intermediate mRNA species after 2, 3, 4, and 5 min of temperature shift-down. Amounts relative to that of time-zero were graphed; *galTKM* (dark circle), *galKM* (gray square), and *galM* (gray triangle).

We transferred a culture of GW20 grown to OD_600_ of 0.6 at 44 to 30°C and continued its growth. We took aliquots of the culture after 5, 10, and 20 min of TSD, prepared RNA, and subjected the RNA to northern blot analysis using the M-probe. The levels of decay intermediates 20 min after TSD (lane 5, Figure 4A), when the temperature of the culture reached 31°C, appeared as much as their levels when the GW20 culture was grown continuously to OD_600_ of 0.6 at 30°C (lane 1, Figure 4A). This indicates that, 20 min after, TSD suffices to restore the full function of RNase E. The same inference could also be made from the appearance of the 16S rRNA band presented as a loading control in Figure 4A. We observed that an RNA band with a molecular weight higher than that of the 16S rRNA appears at the non-permissive temperature (thick arrow, lane 2, Figure 4A). The intensity of this RNA band decreases and that of the 16S rRNA band increases during the TSD period. By 20 min, the ratio of the two bands became comparable to the ratio seen at the permissive temperature. Considering that the RNase E cleavage of the precursor 17S RNA generates 16S rRNA (Li et al., 1999), these results corroborate the abovementioned finding that the function of RNase E is restored by 20 min of TSD.

The sequence of appearance of the decay intermediates during TSD period could be sequential from the 5′ end since, at the earliest time point (5 min) after TSD, the amount of *galTKM* increased the most, followed by the amounts of *galKM* and *galM*, the increase in the latter being hardly detectable (Figure 4A). This suggests that *galTKM* and *galKM* are generated before *galM*. At 10 min after TSD, the amounts of *galTKM* and *galKM* increased 2.7- and 1.6-fold, respectively, relative to their amounts at time-zero (Figure 4C). A slight increase in *galM* amount (about 1.1-fold) was also detected. At 20 min after TSD, the *galM* amount increased 1.8-fold, while the amounts of the other two intermediates, *galTKM* and *galKM*, decreased (Figure 4C). These results further show that *galM* is formed subsequent to the formation of *galTKM* and *galKM*, and their decrease suggests that these longer intermediates could be serving as precursors of *galM*.

During the TSD period, the amounts of both primary transcripts *galETKM* and *galM1* decreased (Figure 4D). Considering that the function of RNase E restores gradually during the TSD period, the gradual decrease in the amounts of *galETKM* mRNA is consistent with the notion that RNase E cleavage of the *galETKM* gives rise to the decay intermediates. The decrease in *galM1* indicates that it is also a substrate of RNase E.

To test if there is a difference in the timing of appearance between *galTKM* and *galKM*, we analyzed the decay intermediates every minute during the first 5-min period after TSD. Temperature gradually fell from 44 to 37°C during the first 5 min (Figure 4B). *galTKM* and *galKM* appeared essentially simultaneously, while the *galM* mRNA amount hardly changed (Figures 4B,E). The simultaneous appearance of *galTKM* and *galKM* indicates that the latter is not produced from the former and that both are independently produced by the RNase E cleavage of *galETKM*. Thus, the appearance of the decay intermediates may not be strictly sequential.

### RNase E Also Cleaves Upstream of the Terminator Hairpin of the *gal* Operon

All the decay intermediates and the primary transcripts *galETKM* and *galM1* have the same terminator hairpin stem-loop structure at the 3′ end (Figure 1B). In the RIT^o^2 mutant, where the function of the stem-loop is impaired, production of these mRNA species was greatly impaired (Figure 1D). The 3′ terminal stem-loop is known to enhance the half-life of mRNA by serving as a road-block to the 3′→5′ exoribonuclease digestion (McLaren et al., 1991; Regnier and Hajnsdorf, 1991; Carpousis et al., 2009). We argued that, to complete the decay of the *galETKM* mRNA, RNase E should cleave upstream of the terminator hairpin of *galM* mRNA to allow 3′→ 5′ exoribonuclease access for digestion of *galM* mRNA.

**FIGURE 5:**
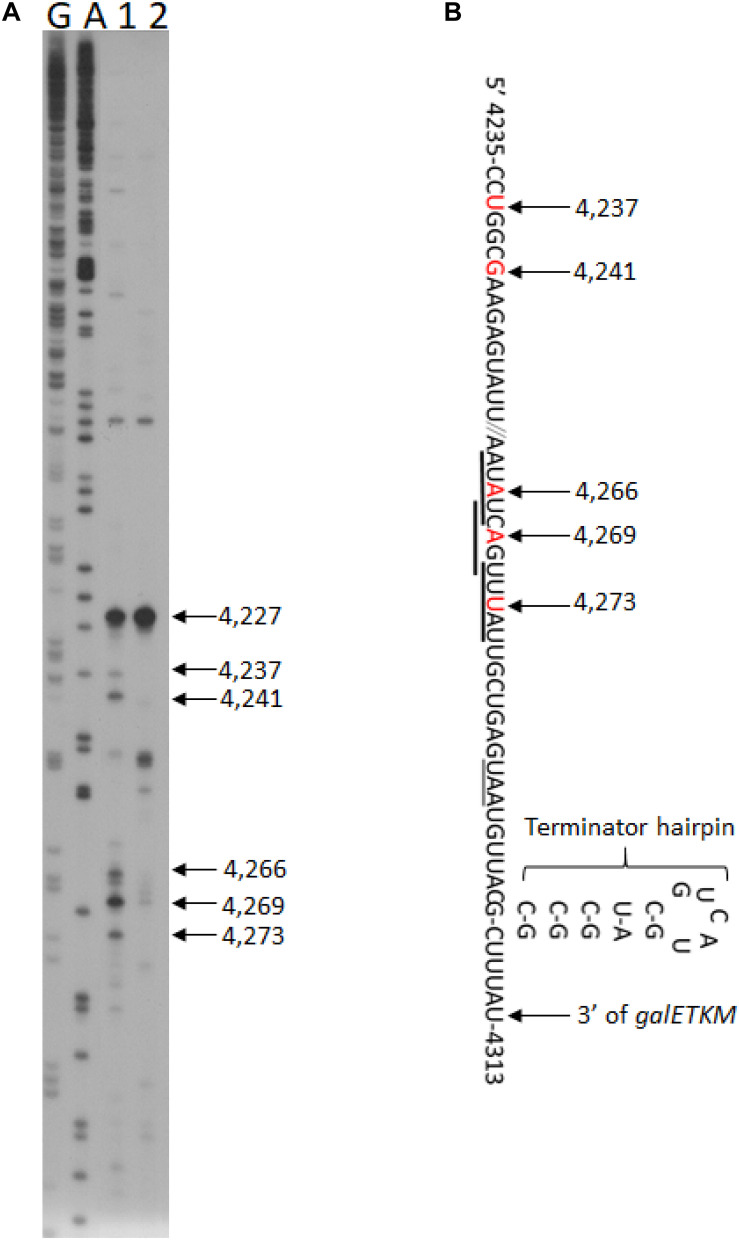
RNase E cleavage upstream of the terminator hairpin of the *gal* operon. **(A)** 5′RACE of RNA in front of the terminator hairpin of *gal* operon isolated from GW20 (*ams1*^*ts*^) cells grown either at the permissive temperature of 30°C (lane 1) or the non-permissive temperature of 44°C (lane 2). The 5′ end at 4,227 is not generated by RNase E cleavage since its intensity did not decrease at the non-permissive temperature. Lanes G and A are DNA sequencing ladders, as in Figure 1E. We note that the 5′ ends located between 4,273 and 4,266 also fall within the consensus sequence for the RNase E cleavage (Belasco, 2017; Chao et al., 2017), but the ends located at 4,241–4,237 seem to deviate from it. Nevertheless, both cleavage clusters would serve the purpose of removing the hairpin, allowing 3′ to 5′ exoribonuclease digestion. **(B)** Nucleotide sequence of mRNA at the end of *galM* presented together with their *gal* coordinates. The nucleotide residue and its *gal* coordinate that becomes the 5′ end after RNase E cleavage are indicated in red. The RNase E cleavage is indicated with a downward arrow. Matches to the consensus sequence for RNase E cleavage are underlined with thick horizontal lines. The translation stop codon of the *galM* gene is also underlined (thin line). Also shown is the terminator hairpin. The cytosine residue at 4,313 is the last residue of the full-length mRNA, *galETKM* (Wang et al., 2019).

To test if there are any RNase E-generated 5′ ends upstream of the terminator hairpin, we performed 5′RACE around the terminator hairpin in GW20 (*ams1*^*ts*^) cells. We found two clusters of 5′ ends at permissive temperature. In one cluster, the ends were at coordinates 4,273, 4,269, and 4,266, and in the other, the ends were at 4,241, 4,237, and 4227 (lane 1, Figure 5A). These 5′ end products became nearly undetectable at non-permissive temperature, except for the product ending at 4,227 (lane 2, Figure 5A). We infer that, barring the end at 4,227, the other five ends were generated by RNase E. The RNase E cleavage sites were at 20–40 nucleotides upstream of the hairpin, which should allow exoribonuclease processing unencumbered by the hairpin (Figure 5B). The origin of the 5′ end at 4,227 remains to be studied.

### Effect of Translation Initiation on the Production of mRNA Intermediates

**FIGURE 6:**
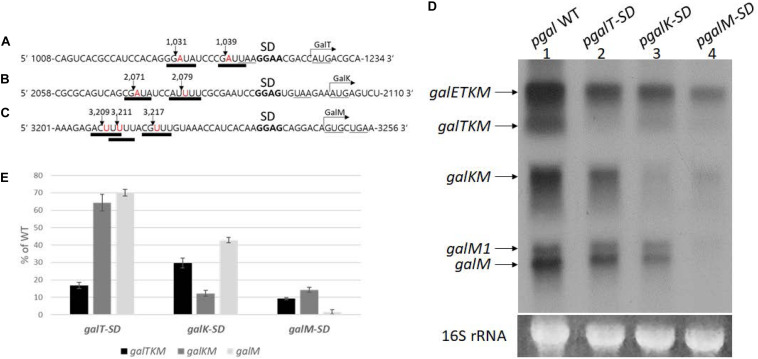
Effect of translation initiation on the production of mRNA intermediates. **(A–C)** Sequence features at cistron boundaries in *gal* mRNA, showing the correspondence of the cleavage positions (inverted arrows) of the samples in Figure 2A to the RNase E consensus cleavage sites (thick underlines). Also highlighted are the putative Shine–Dalgarno sequence (in bold) and initiation (AUG or GUG) and termination (UAA or UGA) codons (underlined). **(D)** Northern blot of *gal* mRNA prepared from MG1655Δ*gal* cells harboring p*gal* (lane 1) or its mutant derivative plasmids, p*galT-SD* (lane 2), p*galK-SD* (lane 3), and p*galM-SD* (lane 4). The blot was probed with the M probe. **(E)** Quantification of each decay intermediate mRNA from **(D)** using the software ImageJ (NCBI, NIH). Note that when mRNA was extracted from cells with p*galT-SD* (lane 2 in **D**), of the three intermediates, *galTKM*, *galKM*, and *galM*, *galTKM* mRNA decreased the most, compared to their levels in cells with the wild-type plasmid p*gal.* Similarly, when the plasmid was p*galK-SD* (lane 3 in **D**), the *galKM* mRNA decreased the most, and the same trend was seen when the plasmid was p*galM-SD* (lane 4 in **D**).

RNase E cleavage is known to have moderate sequence specificity with a degenerate consensus sequence 5′-R(A/G)NW(A/U)UU-3′ (R = A or G, N = any, W = A or U) (Belasco, 2017; Chao et al., 2017). In this sequence, RNase E cleaves between the N and the W residues, leaving the W residue at the 5′ end (Belasco, 2017; Chao et al., 2017). We found that all the 5′ ends of decay intermediates indeed had the W residue (downward arrows) of the consensus RNase E cleavage sequence (underlined) (Figures 6A–C). The 5′ ends of *galTKM* mRNA at 1,031 and 1,039 are 13 and 5 nucleotides upstream from the putative Shine–Dalgarno (SD) sequence of the *galT* gene (Figure 6A). Similarly, in the *galKM* mRNA, the 5′ end is 12 or 20 nucleotides upstream from the putative SD sequence of the *galK* gene (Figure 6B), and in the *galM* mRNA, the 5′ end is 16, 22, or 24 nucleotides upstream from the putative SD sequence of the *galM* gene (Figure 6C).

These results suggest that translation initiation at the beginning of each cistron in *galETKM* could be responsible for the location of the observed RNase E cleavage sites. To test the effect of translation initiation on the observed RNase E cleavage sites, we mutated the putative SD sequences of *galT*, *galK*, and *galM* (bold letters) to their complementary sequences (Figures 6A–C), one SD sequence at a time, and assayed the production of decay intermediates as well as the primary transcripts. These SD mutations were generated in plasmid, p*gal*. The resulting mutant plasmids were called p*galT-SD*, p*galK-SD*, and p*galM-SD*, respectively. We performed northern blot of *gal* mRNA from the three SD mutant plasmid-carrying cells using the M-probe.

Results showed that the primary transcript *galETKM* production decreased gradually, farther down where the SD mutations were in the operon (Figure 6D). The *galETKM* was 60, 55, and 17% of the WT in *galT-SD*, *galK-SD*, and *galM-SD* mutants, respectively. It has been well documented that disruption of the transcription–translation coupling leads to premature Rho-dependent transcription termination (Adhya and Gottesman, 1978). This notion has been rigorously supported in a recent study (Zhu et al., 2019). We reasoned that the premature Rho-dependent transcription termination caused by the SD mutations was responsible for the decreased production of *galETKM*. To test, we performed northern blot of RNA from the SD mutant plasmid-carrying cells that have been treated with the Rho-inhibitor BCM (20 μg/ml for 10 min). The results showed that, upon BCM treatment, *galETKM* increased 20% in cells with p*galT-SD*, 50% in cells with p*galK-SD*, and 250% in cells with p*galM-SD* (Supplementary Figure S3). Thus, in the presence of BCM, SD mutants produced more *galETKM*, suggesting that Rho-dependent transcription termination could be primarily responsible for the decreased production of *galETKM*.

The northern blot of RNA from the SD mutant plasmid-carrying cells (Figure 6D) additionally showed that:

In p*galT-SD*-harboring cells (lane 2, Figure 6D), *galTKM* mRNA decreased to less than 20% of the WT level, while the other two intermediate mRNAs, *galKM* and *galM*, decreased to about 60–70% of the WT level (Figure 6E).
In p*galK-SD*-harboring cells (lane 3, Figure 6D), *galKM* mRNA decreased to 12% of the WT level, while the other two intermediate mRNAs, *galTKM* and *galM*, decreased to about 30–40% of the WT level (Figure 6E).
In p*galM-SD*-harboring cells (lane 4, Figure 6D), *galM* mRNA decreased less than 5% of the WT level, while the other two intermediate mRNAs, *galTKM* and *galKM*, decreased to about 10% of the WT level (Figure 6E).

These results show that, when an intermediate mRNA harbors SD mutation at its 5′ end, its amount is reduced the most than the other two intermediate mRNAs in every case (Figure 6E). The level of decrease of the other two intermediates appeared roughly similar to that of *galETKM* in all of the SD mutants, suggesting that the SD mutations, by decreasing the *galETKM* amount indirectly, caused an overall decrease of the intermediates. We have not correlated mRNA level with protein level, which remains an important task for the future.

### Translation Initiation at the 5′ End of Decay Intermediates Promotes Their Stability

**FIGURE 7:**
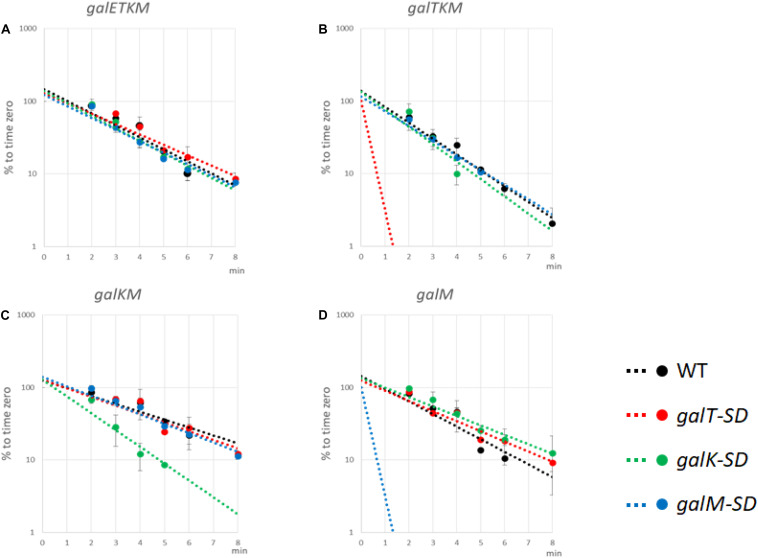
Translation initiation at 5′ end of decay intermediates promotes their stability. The Shine–Dalgarno (SD) mutants as well as wild-type (WT) cells were grown in Luria broth with 0.5% galactose up to OD_600_ of 0.6. Rifampicin (100 μg/ml, final concentration) was added, and thereafter aliquots of the culture were taken at times indicated in the abscissa of the graphs. RNA prepared from the culture samples were subjected to northern blot and probed with M-probe. RNA bands were quantified and plotted against the time of their sampling. Northern blot analyses were repeated at least two times. The decay rate of *galETKM* **(A)**, *galTKM* **(B)**, *galKM* **(C)**, and *galM* **(D)** in WT (black), *galT-SD* (red), *galK-SD* (green), and *galM-SD* (blue) is presented. Note that the decay rate of the primary transcript, *galETKM*, did not change in any of the SD mutants, but the decay rate of an intermediate mRNA decreased significantly only when it harbored an SD mutation at its 5′ end. The dotted lines for *galTKM* in the *galT-SD* mutant [red in **(B)**] and for *galM* in the *galM-SD* mutant [blue in **(D)**] without any experimental points indicate that those RNAs were undetectable even at the earliest sampling time (2 min).

To test the effect of blocking translation initiation further, we measured the half-lives of intermediate mRNA species from cells with the WT or the SD-mutated p*gal* plasmids. MG1655Δ*gal* cells harboring WT or the mutant p*gal* plasmids were grown in LB to OD_600_ of 0.6, at which time rifampicin was added at 100 μg/ml to block new RNA synthesis. Cells were harvested every minute or two, and total RNA was subjected to northern blot analysis using the M-probe. From these blots, we measured the half-lives of the intermediates as well as the full-length *galETKM* mRNA (Figure 7 and Table 1).

**TABLE 1:** Half-life of decay intermediate mRNA (min).

**Strain**	***galETKM***	***galTKM***	***galKM***	***galM***
Wild type	1.82 ± 0.11	1.50 ± 0.08	2.82 ± 0.14	1.86 ± 0.37
*galT-SD*	1.77 ± 0.10	ND	2.10 ± 0.24	1.89 ± 0.01
*galK-SD*	1.76 ± 0.06	1.14 ± 0.10	1.31 ± 0.07	2.29 ± 0.25
*galM-SD*	1.78 ± 0.07	1.52 ± 0.09	2.56 ± 0.38	ND

*Half-life was measured from two independent northern blots. ND, not determined.*

The results are as follows:

*galETKM*: Its half-life in all the SD mutant plasmid-carrying cells was nearly the same as in WT p*gal*-carrying cells, which was 1.82 ± 0.11 min (Figure 7A and Table 1).

*galTKM*: Its half-life in p*galK-SD*- and p*galM-SD*-carrying cells was nearly the same as in WT p*gal*-carrying cells, which was 1.50 ± 0.08 min (Figure 7B and Table 1). However, when the SD mutation was in *galT*, the half-life of *galTKM* decreased to an unmeasurably low value.

*galKM*: Its half-life in p*galT-SD*- and p*galM-SD*-carrying cells was nearly the same as in the WT p*gal*-carrying cells, which was 2.82 ± 0.14 min (Figure 7C and Table 1). However, when the SD mutation was in *galK*, the half-life of *galKM* decreased to 1.31 ± 0.07, significantly below its value in WT p*gal*-carrying cells of 2.82 ± 0.14 (Table 1).

*galM*: Its half-life in p*galT-SD*- and p*galK-SD*-carrying cells was nearly the same as in the WT p*gal*-carrying cells, which was 1.86 ± 0.37 min (Figure 7D and Table 1). However, when the SD mutation was in *galM*, the half-life of *galM* decreased to an unmeasurably low value.

Since the *galETKM* mRNA half-life did not change significantly when the RNA had a defective translation initiation signal in any one of its internal cistrons, *galT*, *galK*, or *galM* (Figure 7A), this suggests that internal translation initiation does not affect RNase E cleavage significantly and thus the decay process of *galETKM*.

Studies with the mutant plasmids revealed that, unlike the SD mutations internal to the operon, when the mutations are present at the 5′ end of an intermediate mRNA, its half-life is specifically reduced (Figures 7B,D). These results suggest that translation initiation at the 5′ end of an intermediate mRNA could be one of the factors that confer stability to the intermediate mRNA. It is also clear that the instability due to SD mutations does not propagate to downstream cistrons, consistent with the view that translation initiation in those cistrons confers stability to them.

### RNase E Cleavage That Results in *galTKM* Generation Requires 5′ Mono-Phosphate

**FIGURE 8:**
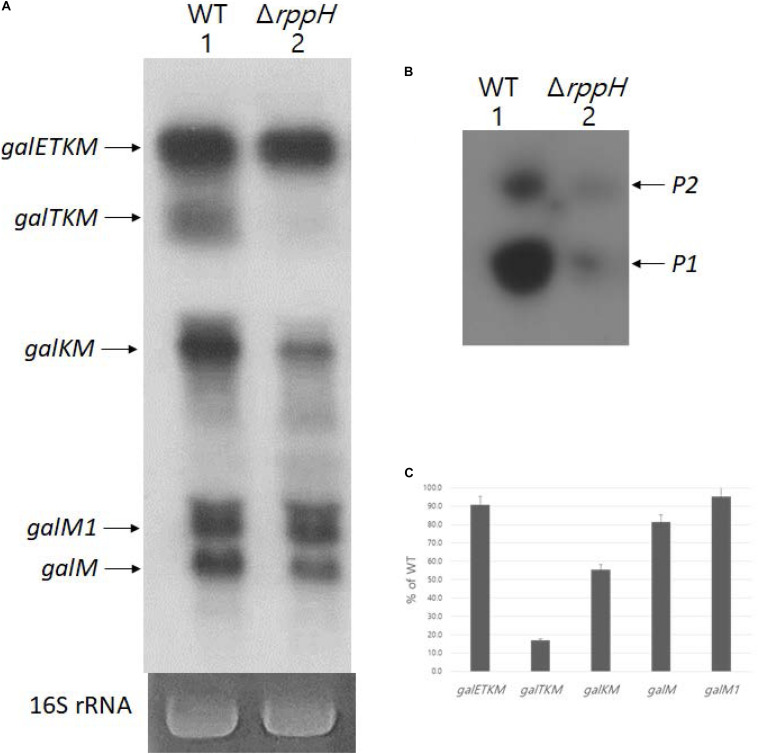
RNase E cleavage that results in *galTKM*generation requires 5’ mono-phosphate. **(A)** Northern blot (M-probed). **(B)** 5’RACE of *gal*mRNA prepared from MG1655Δ*rppH*cells grown at OD_600_ of 0.6. **(C)** Quantification of each decay intermediate mRNA from **(A)** using the software ImageJ (NCBI, NIH).

Di-phosphorylated mRNA 5′ ends are abundant in *E. coli*. RppH removes the β-phosphate from di-phosphorylated mRNA 5′ ends and generates mono-phosphorylated mRNA 5′ ends (Luciano et al., 2017). Thus, in the absence of RppH, the percentage of full-length primary transcripts that are monophosphorylated drops to almost zero as measured by 5′RACE (Deana et al., 2008). To see the generation of the decay intermediates in a situation where most *galETKM* 5′ ends are tri- or di-phosphorylated, we performed northern blot using the M-probe of MG1655Δ*rppH* cells (where the gene for RppH is deleted from the chromosome) grown to OD_600_ of 0.6. The result showed that, while *galETKM* decreased to only 90% of its level in WT, *galTKM* and *galKM* dropped to undetectable and 40% of their level in WT, respectively. The results also showed that *galM* decreased to 80% of WT, but *galM1* hardly at all (Figures 8A,C). Thus, in a situation where most *galETKM* and *galM1* 5′ ends are tri- or di-phosphorylated, mainly the generation of *galTKM* was affected.

Using 5′RACE, we assayed the 5′ end of the *gal* transcript initiated from the two promoters of *gal*, *P1* and *P2* (Adhya and Miller, 1979), in MG1655Δ*rppH*. The 5′RACE results showed that the 5′ ends of *gal* transcripts that are monophosphorylated drastically reduces in MG1655Δ*rppH* compared to their levels in the WT (Figure 8B). Since the 5′RACE assay requires mono-phosphorylated mRNA 5′ end, the 5′RACE assay results indicate that most *galETKM* in MG1655Δ*rppH* are tri- or di-phosphorylated at the 5′ end. Taken together, these results suggest that the reason for the lack of generation of *galTKM* and, to a significant extent, *galKM* is because of the lack of mono-phosphorylation of the 5′ end of *galETKM.* In other words, the 5′ mono-phosphorylated *galETKM* appears to be the precursor of *galTKM* and *galKM*, as was suggested by the results of the TSD experiments (Figure 4). Since *galKM* is still generated in Δ*rppH* cells, albeit in reduced amounts, this indicates that there is a second pathway of generating them which does not depend on a mono-phosphorylated 5′ end.

## Discussion

### The *gal* Operon Produces Two Classes of mRNA: 5′-End-Sharing and 3′-End-Sharing

Northern blot analyses revealed that the *gal* operon produces two kinds of mRNA species that are 5′-end-sharing or 3′-end-sharing, as depicted in Figure 1A. Northern blot of WT *E. coli* cells probed with the E-probe, which hybridizes to the first half of the first gene *galE*, shows the 5′-end-sharing mRNA (lane 1 in Figure 3). However, if the same blot is probed with the M-probe, which hybridizes to the second half of the last gene *galM*, it shows the 3′-end-sharing mRNA (lane 1 in Figure 1D). We investigated how the 5′-end-sharing mRNA species are generated in all of our previous studies (Lee et al., 2008; Wang et al., 2014, 2015, 2019). In this study, we focused on how the 3′-end-sharing mRNA species are generated.

### RNase E Cleavage at Cistron Boundaries

RNase E cleavage at cistron boundaries has been reported in many different operons. Representative examples are in between *dnaG* and *rpoD* (Burton et al., 1983) in the polycistronic *rxc* mRNA (Belasco et al., 1985), in between *papB* and *papA* in the *pap* operon (Baga et al., 1988), and between cistrons of the *his* operon (Alifano et al., 1994b). Recently, using transcriptome analysis, Dar and Sorek demonstrated that RNase E cleavage at the cistron boundaries occur in many different operons in *E. coli* (Dar and Sorek, 2018).

Using the sequence information downstream of the 5′ end of 22,000 RNA generated by RNase E cleavage in *Salmonella* (Chao et al., 2017), we searched for *Salmonella* operons harboring the RNase E-generated 5′ end sequences 40 nucleotides upstream from the initiator codon of internal cistrons. The results showed that 182 operons (out of 881 operons investigated) have RNase E-cleaved 5′ end at cistron boundaries, a few codons upstream from the putative SD sequence of the downstream genes (Supplementary Figure S4). Many operons have RNase E-cleaved 5′ end at multiple cistron boundaries in the same operon (Supplementary Figure S4). Thus, the RNase E cleavages at the cistron boundaries shown in this study appear to be a common practice of mRNA decay of polycistronic mRNA in *E. coli* as well as in *Salmonella*. Of the two activities of RNase E, processing and degradation, generation of stable decay intermediates can also be thought of as a processing event rather than a destructive event.

### Polycistronic *galETKM* mRNA Decays Preferentially Through Cistron Elimination From the 5′ End

Since the initial proposal by Apirion (1973) that mRNA decay in *E. coli* proceeds in 5′→3′ direction, in spite of the lack of any 5′ to 3′ exoribonucleases in *E. coli* (Deutscher, 1985), it is generally believed that mRNA decay in *E. coli* initiates from the 5′ end and proceeds toward the 3′ end (Mackie, 2013; Hui et al., 2014; Mohanty and Kushner, 2016). This is because RNase E has a preference to initiate decay by binding first to the 5′ mono-phosphorylated end. From our limited kinetic analysis of precursor-to-product formation, most of the data could be interpreted as sequential elimination of cistrons from the 5′ end of *galETKM.* A clear exception was also found: About 40% of *galKM* that was dependent on RNase E, but not on 5′ monophosphate formation, was produced (Figures 2A, 4E, 8A). It appears that RNase E can also directly access internal sites within a polycistronic message. As we elaborate below, although overall there is a preference for elimination of cistron from the 5′ end, the process may not be always sequential.

### Use of Two Modes by RNase E in the Elimination of Cistrons From *galETKM* mRNA

**FIGURE 9:**
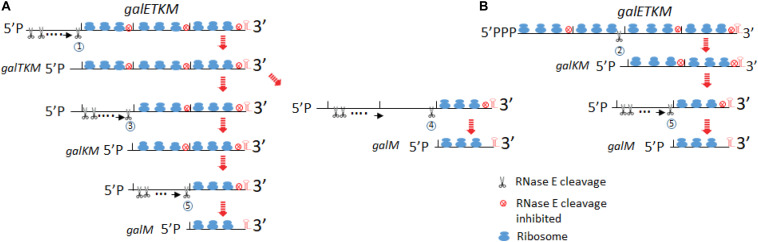
Model for decay of the polycistronic mRNA, *galETKM*. The model depicts two different ways to eliminate a cistron(s) from the 5′ end of *galETKM*. In **(A)**, the *galE* gene is progressively eliminated from the *galETKM* mRNA by the 5′ end-dependent pathway. We propose that RNase E binds to the mono-phosphorylated 5′ end of the *galETKM* mRNA. The RNase E binding causes a cascade of events: (1) inhibition of translation initiation of *galETKM*, (2) receding of already initiated ribosomes from the 5′ end, which would reveal RNase E cleavage sites in the ribosome-free regions of mRNA, and (3) cleavage. Because the newly generated 5′ ends will be mono-phosphorylated, RNase E can again bind and cleave, and the cycle can continue until near the end of the cistron at 1,031 or 1,039, generating *galTKM* by RNase E cleavage labeled 1. By the same process, *galKM* and *galM* (RNase E cleavage labeled 3 and 4, respectively) could be generated. *galM* could also be generated from *galKM* by RNase E cleavage labeled 5. RNase E cleavage 6 would complete the decay of *galETKM*. In **(B)**, we depict the simultaneous elimination of *galE* and *galT* from the *galETKM* mRNA by the direct access pathway. We also propose that the tri-phosphorylated *galETKM* mRNA can stochastically become ribosome-free in the internal cistrons. For example, when the *galE–galT* part becomes ribosome-free, RNase E cleavage labeled 2 at 2,071 or 2,079 directly generates *galKM*. The results in this study suggested that *galKM* could be generated from *galTKM* by RNase E cleavage 3. *galM* could be generated by RNase E cleavage 4 and 5 from *galTKM* and *galKM*, respectively.

Based on the data presented in this study, we propose the following model for the decay of *galETKM* mRNA (Figure 9).

#### Progressive Elimination of *galE* From *galETKM* That Generates *galTKM*

RNase E is a large tetramer and forms a multi-protein complex called the degradosome (Py et al., 1996; Callaghan et al., 2005). Its binding to the 5′ end of mRNA causes steric hindrance to nearby translation initiation (Deana and Belasco, 2005). Inhibition of new translation initiation will make the already initiated ribosomes to retreat away from the 5′ end and expose RNase E cleavage sites which were otherwise to be occluded by translating ribosomes (Figure 9A). When RNase E cleaves the first time, it would generate a new 5′mono-phosphate end, to which RNase E can bind. This cleavage and binding can repeat until the cleavage site approaches the *galT* translation initiation site. The RNase E cleavage labeled as circle 1 would generate *galTKM* (Figure 9A).

In our temperature shift-down experiments, *galTKM* appears to be produced by the gradual shortening of the full-length *galETKM* mRNA during the 5- to 20-min time period (Figure 4A). The trailing of the *galTKM* band (the material between the bands labeled *galETKM* and *galTKM*) is most conspicuous in lane 3 of Figure 4A. The results of 5′RACE indicate that there are two major 5′ ends of *galTKM* located at 1,031 and 1,039, but there are also longer-length species located at ∼890 and 955–980 (Figure 1E), which can be interpreted to represent progressive RNase E cleavage on the *galE* part of *galETKM* during the *galTKM* formation process. We did find a consensus of RNase E cleavage sequences near 890, but none at 955–980. This mechanism is analogous to RNase E scanning activity on RNA from the 5′ end before it cleaves the message (Richards and Belasco, 2019). The generation of *galTKM* was also drastically reduced in Δ*rppH* mutant (Figure 8) in support of the 5′ end-dependent pathway. Thus, the 5′ end-dependent pathway appears to be used exclusively to generate *galTKM* from *galETKM* (Figure 9A).

#### One-Step Elimination of *galET* From 5′-(P)PP–*galETKM* Generates *galKM*

In comparison to *galTKM*, the *galKM* band was less smeary in northern blots (Figure 4A). Only two closely spaced ends of *galKM* at 2,071 or 2,079 were also revealed by 5′RACE (Figure 1E). The appearance of *galTKM* and *galKM* at the same time and the RppH-independent accumulation of *galKM* through direct-entry processing of a triphosphorylated *galETKM* precursor suggest that RNase E generates *galKM* directly from full-length *galETKM* and not from intermediate length precursors, as observed for *galTKM*. Since *galTKM* and *galKM* appear at the same time, they are most likely produced independently from different *galETKM* RNAs. Alternatively, it is also possible that, once it has formed, *galTKM* is rapidly cleaved to produce *galKM* in a step that is not rate limiting.

Since a significant amount of *galKM* is generated in Δ*rppH* cells (Figure 8), it indicates that the direct access pathway was responsible for the generation. Since the level of production was different in the WT and Δ*rppH* cells, this indicates that both the direct access (by RNase E cleavage labeled as circle 2 in Figure 9B) and the 5′ end-dependent (by the circle 3 RNase E cleavage in Figure 9A) pathways are employed to generate *galKM*. Note that, in *E. coli*, ∼35–50% of each mRNA is di-phosphorylated, and the rest is mono-phosphorylated at the 5′ end (Luciano et al., 2017), and the direct access pathway of mRNA decay has been demonstrated as a major pathway for mRNA decay in *E. coli* (Clarke et al., 2014).

#### Elimination of a Cistron(s) to Generate *galM*

From the temperature shift-down experiment, we propose the following pathways for *galM* mRNA generation. By the sequence of appearance, *galM* could be generated either by the simultaneous removal of the *galTK* part from *galTKM* (the RNase E cleavage labeled as circle 4 in Figure 9A) or by removing the *galK* part from *galKM* (the cleavage labeled as circle 5 in Figure 9A). The decrease of *galTKM* and the increase of *galM* from 10 to 20 min time points in one hand and relatively no change in *galKM* amount on the other may suggest that *galM* is derived directly from *galTKM* by the cleavage 4, but the alternate pathway, indicated by the cleavage 5, such that *galM* is primarily derived from *galKM*, which is derived from *galTKM*, cannot be eliminated (Figure 9A). *galKM* amounts are not expected to change if the rate of decay of *galTKM* to *galKM* and *galKM* to *galM* is similar. The generation of *galM* in Δ*rppH* cells does not necessarily indicate that it is produced by direct access if the precursors are *galTKM* and/or *galKM*, which have a mono-phosphorylated 5′ end. This indicates that *galM* is derived from the 5′ end-dependent pathway. The smearing RNA below the *galKM* band in the first 5 min (Figure 4B) or at 10 min (Figure 4A) of TSD suggests that the *galK* part of the *galKM* mRNA could be progressively removed from the 5′ end (as indicated in the cleavage 5). However, the smearing does not extend all the way down to the *galM* band (Figure 4B), suggesting that the progressive elimination of *galK* proceeds to a certain point of *galK* before RNase E cleaves at 3,209–3,217 (Figure 2B) to generate *galM*.

#### Elimination of *galM*

Elimination of *galM* is the last step in the sequence of cistron elimination, completing the decay process of *galETKM* mRNA. The *galM* band also smears to lower-size species to some extent, indicating that it decays progressively (Figure 4B). However, RNase E could also directly access the cleavage sites at 4,266–4,273.

### Failure in Translation Initiation at the 5′ End of Polycistronic mRNA Initiates mRNA Decay

Our results here demonstrate that interfering with translation initiation at the 5′ end of each intermediate mRNA greatly increases the message decay rate (Figure 7 and Table 1). Because translating ribosomes inhibit RNase E cleavage (Deana and Belasco, 2005) and RNase E cleaves mRNA regions free of ribosome (Iost and Dreyfus, 1995), the “absence of ribosome” seems obligatory for the cleavage. Inhibiting translation initiation seems the most reasonable way to generate ribosome-free regions within a message. Creating ribosome-free regions within an ORF undergoing translation other than by inhibiting translation initiation would lead to the production of truncated proteins, which would be wasteful (Keiler et al., 1996; Muto et al., 1996; Hayes et al., 2002). Thus, RNase E cleavage on *galTKM* or *galKM* to generate *galM* is most likely preceded by inhibition of translation initiation at the 5′ end.

The same decay strategy could be applied to the primary (full-length) polycistronic mRNA. Considering that both of the 5′ end-dependent and direct access pathways work on the primary mRNA, *galETKM*, it is likely that the phosphorylation status of 5′ end influences which pathway RNase E would take to initiate the cleavage. Aside from occasional stochastic failure to initiate translation, there could be formation of a stem-loop structure as in the 5′ UTR (Arnold et al., 1998), other secondary structures, or sRNA binding that could inhibit translation initiation at the 5′ end of many mRNAs. For example, sRNA Spot 42 binding to the 5′ end of one of the intermediate *gal* mRNA, *galKM* (mK2), expedites the decay of *galKM* (Wang et al., 2015). Even proteins such as RNase E itself could inhibit translation initiation (Deana and Belasco, 2005). We propose that it is these translation initiation-inhibiting factors at the 5′ end of polycistronic mRNA that induce cleavage by making the RNase E cleavage sites ribosome-free. Our experiments also revealed that the cistron elimination did not proceed to the downstream cistrons possibly because of their translation initiation signals.

In all the cartoons of Figure 9, a cistron was eliminated when it was the 5′-most cistron and ribosome-free. This was evident in experiments of Figure 6 where translation initiation signals were mutated. For example, in the case of the *galT-SD* mutant, whatever pathway generates *galTKM* from *galETKM*, the intermediate is eliminated selectively if it did not have a translation initiation signal of *galT*. However, *galTKM* was not preferentially eliminated, although it was the 5′-most cistron when it had the *galK-SD* mutation (Figure 6). Direct entry of RNase E into the ribosome-free *galK* region should have eliminated the *galTKM* intermediate together with *galKM*, but apparently that did not happen. In other words, the absence of translation, although necessary, is not sufficient for decay.

In sum, even in a four-cistron mRNA, there can be progressive and one-step elimination of cistrons and no elimination of internal cistrons even in the absence of translation. Although the picture is complex, vulnerability of the 5′-most cistrons appears to be the most consistent theme of the present study, in line with the general thinking that decay proceeds in the 5′→3′ direction.

### Future Studies on *gal* mRNA Decay

Intermediates of *gal* mRNA other than those described here are also produced by agents such as Spot 42 small RNA and transcription terminator factor. For example, in MG1655Δ*spf* cells, where the gene for Spot 42 is deleted from the chromosome, the production of *galKM* mRNA is reduced compared to that seen in the WT, and this is because Spot 42 expedites the decay of the *galKM* mRNA (Wang et al., 2015). Based on these results, we propose that Spot 42 binding might expedite the decay of those *gal* mRNA species, such as *galETKM*, *galTKM*, and *galKM*, that harbor the Spot 42 binding site at the *galT–galK* cistron junction. We are currently testing this prediction. We are also currently investigating whether in the generation of the 5′ end of *galKM* there is a relationship between the RNase P cleavage at 1,764 and 1,777 (Wang et al., 2014) and the RNase E cleavage at 2,071 and 2,079 (Figure 1E).

## Data Availability Statement

All datasets presented in this study are included in the article/Supplementary Material.

## Author Contributions

HL designed the research. HJ, CK, MN, and YL performed the research. HJ, CK, MN, YL, XW, DC, and HL analyzed the data. DC and HL wrote the manuscript. All authors contributed to the article and approved the submitted version.

## Conflict of Interest

The authors declare that the research was conducted in the absence of any commercial or financial relationships that could be construed as a potential conflict of interest.

## Supplementary Material

Supplementary Figure 1 Newly found internal promoter *M* and the transcript *galM1*.

Supplementary Figure 2 Effect of bicyclomycin, the Rho-inhibitor, on the production of *gal* mRNA.

Supplementary Figure 3 Production of *galETKM* in the Shine–Dalgarno mutants in the presence of bicyclomycin.

Supplementary Figure 4 Search of *Salmonella* operon harboring the 5′ end of mRNA at cistron boundaries.

Supplementary Table 1 A list of primers used in this study.
