# Supplementary material for: Translation Initiation Control of RNase E-Mediated Decay of Polycistronic gal mRNA
Source: Front Mol Biosci. 2020 Nov 6;7:586413. doi: 10.3389/fmolb.2020.586413 (PMC7681074; doi:10.3389/fmolb.2020.586413)
Supplement: Supplementary Figure 1 — Newly found internal promoter M and the transcript galM1. [file Table_1.docx]

Supplementary Information for

**Translation initiation control of RNase E-mediated decay of polycistronic *gal* mRNA**

**Authors:** Heung Jin Jeon^1, 4^, Changjo Kang^1^, Monford Paul Abishek N^1^, Yonho Lee^1^, Xun Wang^2^, Dhruba K. Chattoraj^3^, Heon M. Lim^1^*

*Corresponding Author:

Heon M. Lim Email: hmlim@cnu.ac.kr

**This PDF file includes:**

Figures S1 to S3

Tables S1

**Translation initiation control of RNase E-mediated decay of polycistronic *gal* mRNA**

**Jeon et al.**

**Supplementary Figure S1**

**Newly found internal promoter *M* and the transcript *galM1***


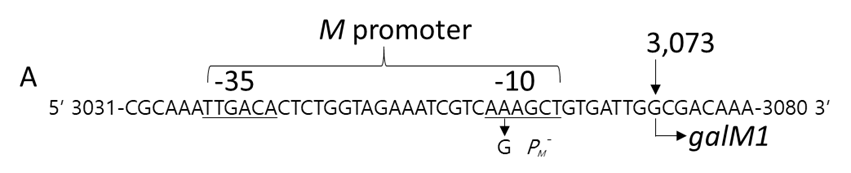
We searched upstream of the 5’ end of *galM1* for a consensus promoter sequence and found 5’-TTGACA-3’, a perfect match to the -35 region of the promoter for sigma-70 RNA polymerase, at 3,037 of the *gal* coordinate. Seventeen nucleotide downstream of the -35 region, we found the -10 region 5’-AAAGCT-3’ **(**Fig. A**)**. We changed the second adenine residue (3,061) of the -10 region to guanine, creating the -11G *M* promoter (*P_M_^-^*) mutant that significantly downregulates sigma-70 promoter activity (Lim et al. 2001). Northern blot with the M3-probe showed that the *galM1* almost disappeared in the *P_M_^-^* mutant (lane 2 in Fig. S1B), demonstrating that the *galM1* mRNA is the transcript from the internal M promoter of *gal.* Transcription from the *M* promoter starts at 3,073.


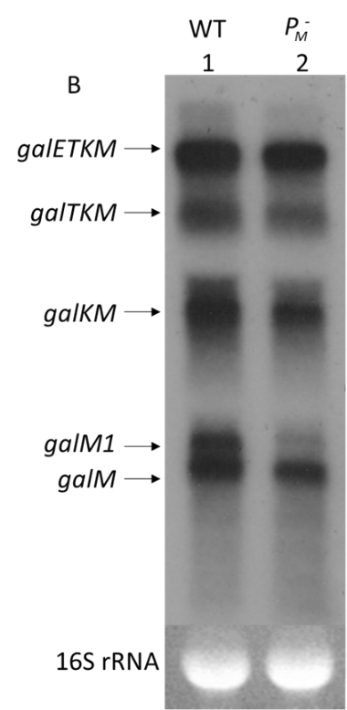


**Fig. S1** (A) Nucleotide Sequence around the *M* promoter (*P_M_*). (B) Northern blot of the *gal* mRNA in MG1655Δ*gal* harboring the plasmid p*gal* (lane 1), MG1655Δ*gal* harboring the plasmid p*gal-P_M_^-^* (lane 2).

**Reference**

Lim, H.M., Lee, H.J., Roy, S. and Adhya, S. (2001) A "master" in base unpairing during isomerization of a promoter upon RNA polymerase binding. *Proceedings of the National Academy of Sciences of the United States of America*, **98**, 14849-14852

**Translation initiation control of RNase E-mediated decay of polycistronic *gal* mRNA**

**Jeon et al.**

**Supplementary Figure S2**

**Effect of bicyclomycin (BCM), the Rho-inhibitor, on production of the *gal* mRNA**

WT MG1655 cells were grown in LB with 0.5 % galactose to OD_600_ of 0.6. BCM was added to the culture 20, 40 and 80 µg/ml of BCM (final concentration). The cultures were continued for additional 10 min. RNAs were prepared form the cultures, and subjected to the Northern blot analysis. Results showed that production of all the 5’-sharing *gal* mRNA species were gradually decreased with increasing concentration of BCM (Fig. S2A). In the presence of 80 µg/ml BCM, the *galETKM, galETK, galET, galE1,* and *galE* decreased to 34, 06, 09, 7 and 42 % of no BCM (Fig. S2B). Some of the *gal* transcriptions, if not terminated by Rho-dependent transcription termination at the end of the operon, continue to the *gpmA* gene, which is the downstream gene of *gal*. These transcriptions are terminated at the end of the *gpmA* gene, and produce an mRNA species of 5.6 kb in size shown above the full-length *gal* mRNA, *galETKM* (A) (Wang et al. 2019).


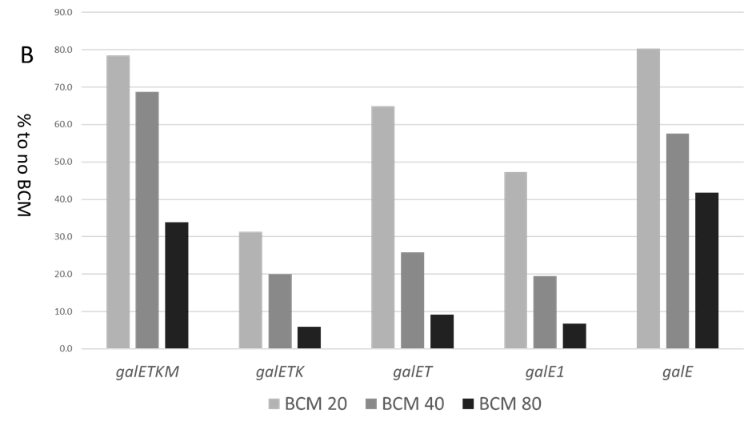

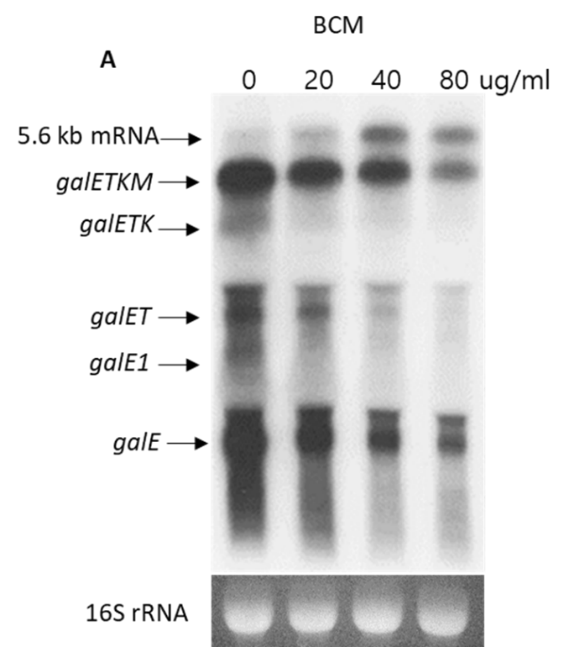


**Fig. S2 (A)** Northern blot of the *gal* mRNA in MG1655 WT cells grown for 10 min in the presence of indicated concentration of BCM. This northern blot was probed the E probe that hybridizes to the first half of the *galE* gene of the *gal* operon. (B) Quantification of each mRNA band shown in (A).

**Reference**

Wang, X., N, M.P.A., Jeon, H.J., Lee, Y., He, J., Adhya, S. and Lim, H.M. (2019) Processing generates 3' ends of RNA masking transcription termination events in prokaryotes. *Proceedings of the National Academy of Sciences of the United States of America*.

**Translation initiation control of RNase E-mediated decay of polycistronic *gal* mRNA**

**Jeon et al.**

**Supplementary Figure S3**

**Production of *galETKM* in the SD mutants in the presence of BCM**

It has been well documented that disruption of transcription-translation coupling leads to Rho-dependent transcription termination (Adhya Gottesman 1978, Zhu et al. 2019). We reasoned that mutations at the translation initiation site of the cistrons in the SD mutants could lead to disruption of the coupling between transcription and translation. This would lead to Rho-dependent transcription termination, which would cause less transcription reaching the end of the operon. Thus, premature transcription termination provoked by the SD mutations would lead to decreasing amount of the full-length mRNA, *galETKM*. To test the notion, we treated the SD mutants with the Rho inhibitor, BCM, expecting that production of *galETKM* in the SD mutants would restore to that of WT.

MG1655Δ*gal* cells harboring, *pgal* (WT), *pgalT-SD*, *pgalK-SD*, and *pgalM-SD* plasmid were grown in LB with 0.5 % galactose to OD_600_ of 0.6. Each culture was divided into two halves. In one set of cultures, BCM was added 20 µg/ml (final concentration). Both culture sets were continued for additional 10 min. RNAs were prepared form the cultures, and subjected to northern blot probed with the M-probe (A). Quantification of the *galETKM* RNA band (B) showed that, in the BCM treated culture set, *galETKM* production increased 120 % in *galT-SD*, 150 % in *galK-SD*, and 250 % in *galM-SD*.


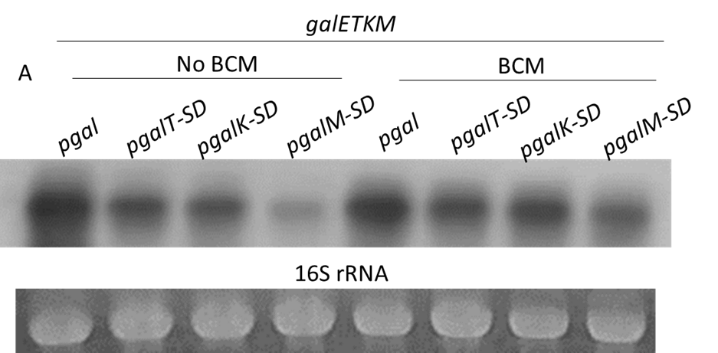

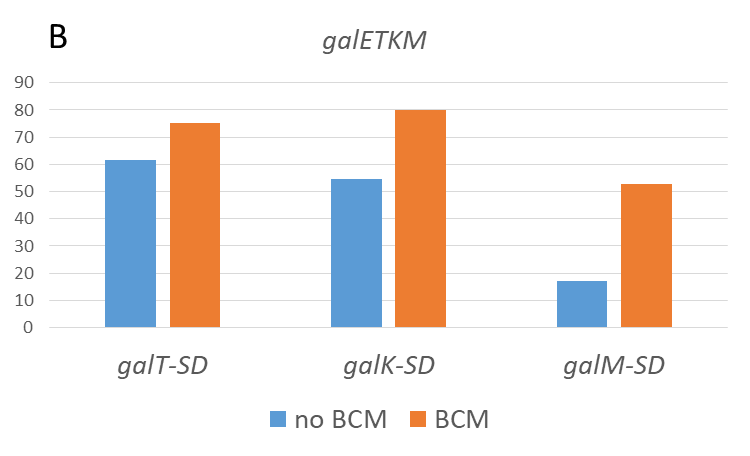


**Fig. S3** (A) Northern blot of *galETKM* mRNA in the SD mutants in absence (left set of 4) and presence of 20 µg/ml of BCM (right set of 4). (B) Quantification of *galETKM* is presented as % of *pgal* (WT) in each set.

**References**

Adhya, S. and Gottesman, M. (1978) Control of transcription termination. *Annual review of biochemistry*, **47**, 967-996

Zhu, M., Mori, M., Hwa, T. and Dai, X. (2019) Disruption of transcription-translation coordination in Escherichia coli leads to premature transcriptional termination. *Nature microbiology*.

**Translation initiation control of RNase E-mediated decay of polycistronic *gal* mRNA**

**Jeon et al.**

**Supplementary Table S1. Primers used in this study.**

| **Primer Name** | **Primer sequence 5′ to 3′** | **Use** |
| --- | --- | --- |
| E1-for | ATGAGAGTTCTGGTTACCGGTGGTA | Northern E probe generation |
| E2-rev | TGGGCTTTTTGCAGATCGGTGAGGA |  |
| M1-for | GGTGAATATGACTAATCACGTCTATTT | Northern M probe generation |
| M2-rev | TTACTCAGCAATAAACTGATATTCCGT |  |
| 5SF | GAGAGTAGGGAACTGCCA | 5′ RACE PCR primer |
| T2-rev | CTGACCGTGCGGATGCGGGTTAGAG | 5`RACE PCR primer  (3.2kb *galTKM* mRNA) |
| K2-rev | AGCCTACAAACTGGTTTTCTGCTTCC | 5`RACE PCR primer  (2.3kb *galKM* mRNA) |
| M2-1-rev | CATCTGAACTCAGGGCAAACA | 5`RACE PCR primer  (1.1kb *galM* mRNA) |
| EText-rev | AGAATCCATTGCCCGGTGAG | 5`RACE extension primer  (3.2kb *galTKM* mRNA) |
| TKext-rev | ATGGTGTGAGTGGCAGGGTA | 5`RACE extension primer  (2.3kb *galKM* mRNA) |
| KMext-rev | TGCCAGTGCGGGAGTTTCGT | 5`RACE extension primer  (1.1kb *galM* mRNA |
| mM1ext-rev | ATAAAGCCCTCATGACGAGG | 5`RACE PCR primer /  5`RACE extension primer  ( galM terminator hairpin ) |
| -73-EcoRI-for | GGCGAATTCAATTCTTGTGTAAACGATTCC | *gal* mutagenesis, and cloning |
| Gal-HindIII-F | CACCGTTTATGGCGATCAGCCC |  |
| Gal-MluI-R | GCGTTTTCAGTCAGTATATGACG |  |
| Gal-MluI-F | CATGAACTGGACCCGATCGTGG |  |
| Gal-BamHI-R | CACCCCAGGCTTTACACTTTATGC |  |
| T-NO_SD_R | GGGATTAGGGAGTGGATGGCGTGACT |  |
| K-NO_SD_R | CTTACGAGGGGATTCGCGAAAATGGATAT |  |
| M-NO_SD_R | GTCCTGGAGGTTGTGATGGTTTACAAACG |  |
